# Discovering Biomarkers and Pathways Shared by Alzheimer’s Disease and Ischemic Stroke to Identify Novel Therapeutic Targets

**DOI:** 10.3390/medicina55050191

**Published:** 2019-05-22

**Authors:** Md. Rezanur Rahman, Tania Islam, Md. Shahjaman, Toyfiquz Zaman, Hossain Md. Faruquee, Mohammad Abu Hena Mostofa Jamal, Fazlul Huq, Julian M. W. Quinn, Mohammad Ali Moni

**Affiliations:** 1 Department of Biochemistry and Biotechnology, School of Biomedical Science, Khwaja Yunus Ali University, Sirajgonj 6751, Bangladesh; toyfiquz19@gmail.com; 2 Department of Biotechnology and Genetic Engineering, Islamic University, Kushtia 7003, Bangladesh; taniaislam1304@gmail.com (T.I.); faruqueebt2008@gmail.com (H.M.F.); jamalbtg@gmail.com (M.A.H.M.J.); 3 Department of Statistics, Begum Rokeya University, Rangpur 5400, Bangladesh, shahjaman_brur@yahoo.com; 4 Discipline of Pathology, School of Medical Sciences, Faculty of Medicine and Health, The University of Sydney, Sydney, NSW 2006, Australia; fazlul.huq@sydney.edu.au; 5 Bone Biology Division, Garvan Institute of Medical Research, Darlinghurst, NSW 2010, Australia; j.quinn@garvan.org.au

**Keywords:** Alzheimer’s disease, ischemic stroke, drug targets, biomarker signatures, differentially expressed genes, protein–protein interaction, protein–drug interactions

## Abstract

*Background and objectives*: Alzheimer’s disease (AD) is a progressive neurodegenerative disease that results in severe dementia. Having ischemic strokes (IS) is one of the risk factors of the AD, but the molecular mechanisms that underlie IS and AD are not well understood. We thus aimed to identify common molecular biomarkers and pathways in IS and AD that can help predict the progression of these diseases and provide clues to important pathological mechanisms. *Materials and Methods*: We have analyzed the microarray gene expression datasets of IS and AD. To obtain robust results, combinatorial statistical methods were used to analyze the datasets and 26 transcripts (22 unique genes) were identified that were abnormally expressed in both IS and AD. *Results*: Gene Ontology (GO) and KEGG pathway analyses indicated that these 26 common dysregulated genes identified several altered molecular pathways: Alcoholism, MAPK signaling, glycine metabolism, serine metabolism, and threonine metabolism. Further protein–protein interactions (PPI) analysis revealed pathway hub proteins PDE9A, GNAO1, DUSP16, NTRK2, PGAM2, MAG, and TXLNA. Transcriptional and post-transcriptional components were then identified, and significant transcription factors (SPIB, SMAD3, and SOX2) found. *Conclusions*: Protein–drug interaction analysis revealed PDE9A has interaction with drugs caffeine, γ-glutamyl glycine, and 3-isobutyl-1-methyl-7H-xanthine. Thus, we identified novel putative links between pathological processes in IS and AD at transcripts levels, and identified possible mechanistic and gene expression links between IS and AD.

## 1. Introduction

Alzheimer’s disease (AD) is a progressive neurodegenerative disease causing severe dementia and cognitive decline in elderly people. There are now 5.5 million AD sufferers in USA and the number is predicted to rise to 13.8 million by 2050 [1]. The pathobiology of AD involves the formation of amyloid plaques and tangles in neurofibrils [2] which may disrupt neuron function, although it is controversial as to whether this is causative. Ischemic strokes (IS) is usually estimated as the second highest cause of death worldwide, and is predicted to rise to 81.1 million incidents by 2040 [3]. When considered separately from other cardiovascular diseases, stroke accounts for killing nearly 133,000 people a year in the USA [4]. The risk of IS (and AD) increases after age 55, although it can occur at any age. Even if in a minority of cases (less than 5%), AD can be genetically determined. In these cases, defined as familial AD, symptoms show much earlier in life if compared to sporadic AD. Thus the prevalence, mortality, and associated morbidity make AD and IS a major health care burden globally [4].

**Figure 1:**
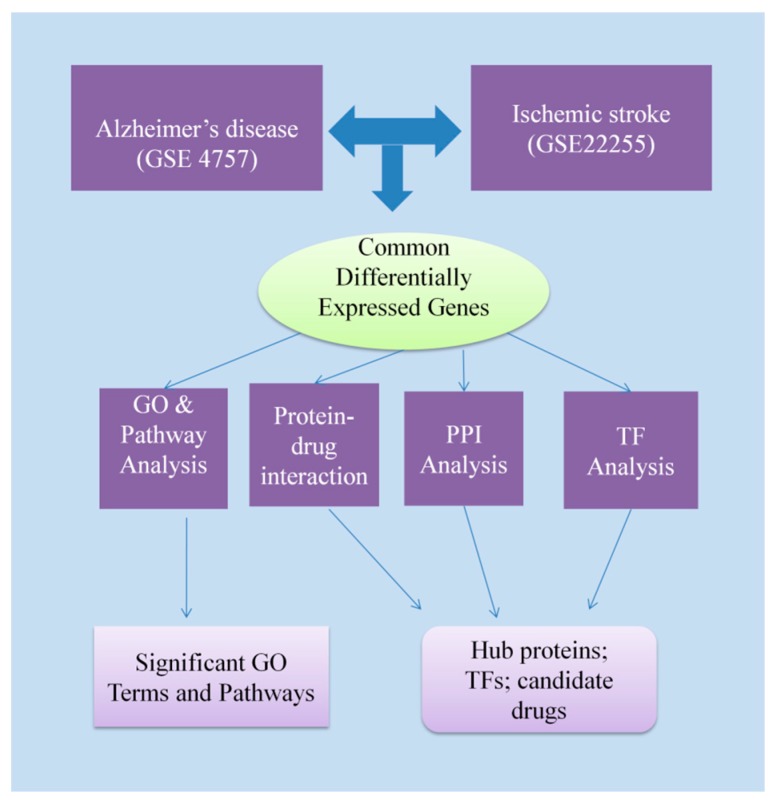
The integrative approach employed in the present study. The gene expression data of Alzheimer’s disease (AD) and ischemic stroke (IS) were obtained from the Gene Expression Omnibus. The datasets were analyzed by a combinatorial statistical approach to identify the differentially expressed genes (DEGs). Then, common DEGs between the AD and IS were identified by these methods. The common DEGs between AD and IS (i.e., genes dysregulated in both AD and IS) were subjected to Gene Ontology (GO) and pathways enrichment analyses to obtain significant GO terms and pathways. The proteins encoded by the common DEGs were also analyzed by protein–protein interaction (PPI) analysis to identify hub proteins. The transcriptional regulators of the DEGs were identified, and the protein–drug interactions analysis was performed to identify compounds that may block pathway hub proteins. TF: Transcription factor.

There is convincing evidence for epidemiological and pathological links between AD and IS from population-based studies which revealed that AD is a highly significant risk factor for IS [5,6], and vice versa [7]. Clearly, both involve neurological damage, and it is possible that there are shared pathological mechanisms underlying both conditions [8,9,10]. Several studies have tried to identify genetic links between IS and AD, for example, by genome-wide association studies; these have identified common genetic elements, mainly single nucleotide polymorphisms in IS and AD that point to at least some shared element in their pathogenesis [10,11,12,13,14,15,16,17,18], but the significance of these is currently uncertain. Molecular components that are commonly dysregulated in both diseases is, to date, very limited in AD and IS. However, candidate biomarkers, such as differentially expressed genes (DEGs), have been identified using transcriptomic datasets from RNA-seq and microarray studies [14,15,18]. Therefore, to capitalize on the availability of these datasets, we have employed a systems biology approach to identify dysregulated genes and molecular pathways that are common to AD and IS affected tissues. We have used findings from such analyses to integrate the differentially expressed genes (DEGs) in AD and IS tissues with interaction networks using the following approaches: (i) A protein–protein interactions (PPI) network of the proteins encoded by the common DEGs; (ii) identification of transcriptional and post-transcriptional regulatory components of the common DEGs; (iii) protein–drug interaction networks to screen potential drugs. This comprehensive systems biology pipeline thus allows us to explore possible critical pathway hub protein transcription factors (TFs), transcripts of interest, and to provide potential biomarker signatures useful for disease assessment and further biological research. We can also use these insights to identify drug binding partners to some of the hub protein that may indicate new therapies if the proteins prove to have important pathological influences on AD and IS (Figure 1).

## 2. Materials and Methods

### 2.1. Identification of Differentially Expressed Genes in AD and IS

The microarray gene expression high-throughput analysis datasets for IS- and AD-affected tissues were obtained from the NCBI-GEO database [19]. The IS dataset was accession number GSE22255, which was generated using peripheral blood mononuclear cells (PBMCs) from 20 IS patients and 20 sex- and age-matched controls using Affymetrix microarrays. The AD dataset was GEO accession number GSE4757 dataset of laser-capture microdissected brain tissue (entorhinal cortex layer 2 stellate island neurons) containing approximately 1000 cells attached to neurofibrillary tangles from mid-stage AD patients, and matched neurons in normal tissues as controls. The datasets were first analyzed in the Bioconductor environment implemented in R (version R × 64 3.4.1) to identify DEGs in the IS and AD data relative to their respective matched controls. Firstly, the gene expression dataset was normalized by log_2_ transformation and combinatorial statistical methods using the Limma package in R, Kruskal-Wallis, and Student’s *t*-test in hypothesis testing, with Benjamini–Hochberg correction to control the false discovery rate. A *p*-value < 0.05 and absolute log_2_ fold change (FC) >=1 was regarded as threshold criteria for significant DEGs of interest. 

### 2.2. Gene Ontology and Pathway Enrichment Analysis

Gene overrepresentation analyses were performed to identify Gene Ontology (GO) (i.e., biological process, cellular component, and molecular functions) and KEGG pathways using NetworkAnalyst [20]. An adjusted *p*-value < 0.05 was considered significant for all the enrichment analyses. 

### 2.3. Protein–Protein Interaction Analysis

We used the STRING protein interactome database [21] to construct PPI networks of the proteins encoded by the identified DEGs. We set a medium confidence score (400) to construct the PPI, since the number of DEGs was low. Network analysis was performed using the NetworkAnalyst online resource [20].

### 2.4. Identification of Transcriptional and Post-Transcriptional Regulator Components

We used Enrichr [22] to identify regulatory TFs which regulate DEGs of interest at the transcriptional level using TRANSFAC database [23]. A *p*-value < 0.05 was considered significant for the analysis.

### 2.5. Protein–Drug Interactions Analysis

The protein–drug interaction was analyzed using DrugBank database (Version 5.0) [24] to identify potential drugs to be proposed in the AD. The protein–drug interactions analysis was performed using NetworkAnalyst [19]. 

## 3. Results

### 3.1. Identification of Differentially Expressed Genes Common to AD and IS

**Figure 2:**
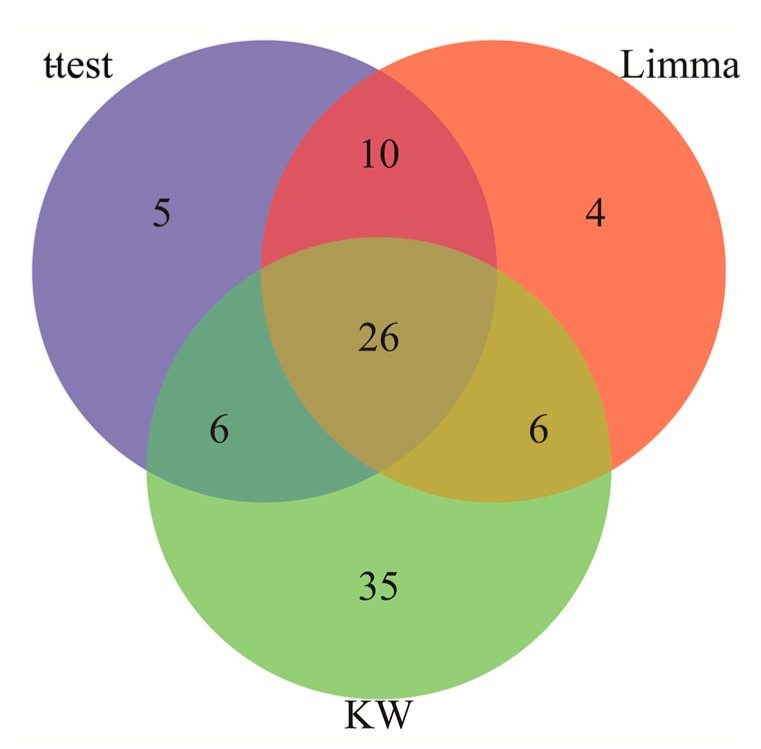
The gene expression datasets of Alzheimer’s disease (AD) and ischemic stroke (IS) were analyzed by combinatorial statistical approaches comprising Limma, Kruskal-Wallis (KW), and *t*-tests (*t*-test) to identify the common differentially expressed genes (DEGs) between the AD and IS. Twenty-six transcripts (22 unique genes) identified by all three approaches were regarded as the DEGs common to IS and AD.

**Figure 3:**
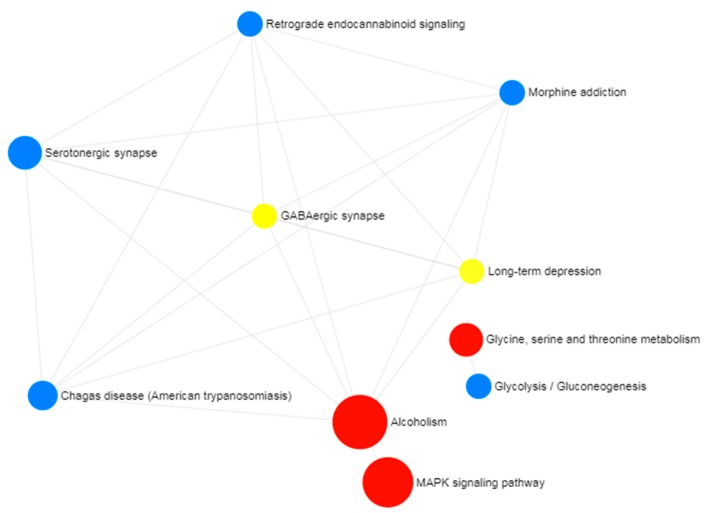
The significant molecular pathways enriched by the common differentially expressed genes in Alzheimer’s disease and ischemic stroke. The molecular pathways alcoholism, MAPK signaling pathway, and glycine, serine, and threonine metabolism pathways were statistically significant (*p*-value < 0.05).

The IS and AD gene expression microarray datasets were analyzed and for both datasets significant DEGs were identified using applied combinatorial statistical methods. Twenty-six transcripts (representing 22 unique genes) were identified as common between the IS DEGs and AD DEGs (Figure 2). This set of identified genes was subjected to gene set enrichment analysis to obtain biological process, molecular function, and cellular component enrichment that the genes participate in. The enriched biological processes (BP) were regulation of axonogenesis, learning or memory, brain development, behavior, aging, and others shown in Table 1. The significant molecular function of the DEGs common to IS and AD were phosphoric ester hydrolase activity, 3′,5′-cyclic nucleotide phosphodiesterase activity, phosphatase activity, hydrolase activity, acting on ester bonds, antiporter activity, and hormone binding (Table 1). The significant cellular pathways were identified as neuron projection, cell projection part, and axon and cell projection (*p*-value < 0.05) (Table 1). The altered molecular pathways identified included alcoholism, MAPK signaling pathway, and glycine, serine, and threonine metabolism (Figure 3).

### 3.2. Identification of Hub Proteins

**Figure 4:**
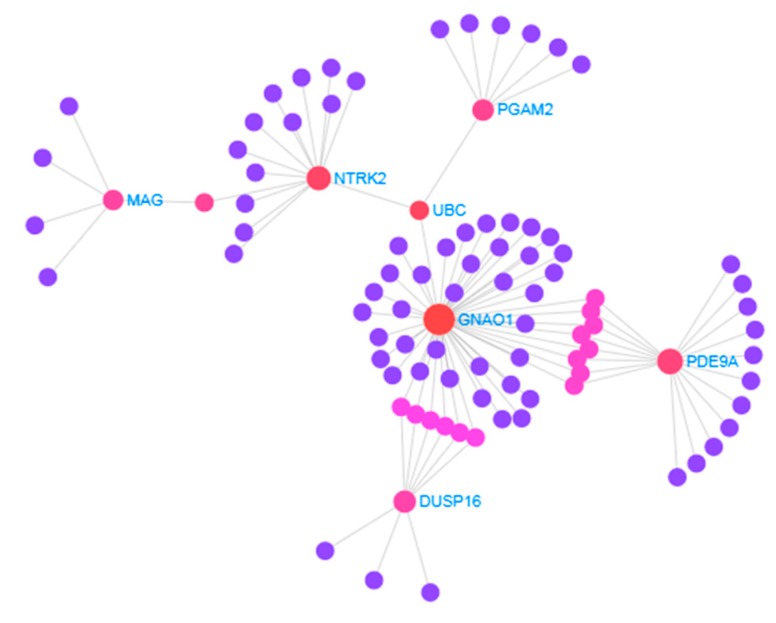
The protein–protein interaction of the common differentially expressed genes (DEGs) with other genes was retrieved from the STRING database. The PPI network consists of 94 nodes (7 nodes from the common DEGs) and 97 edges. The nodes indicate the DEGs and the edges indicate the interactions between two genes.

Firstly, a protein–protein interactions (PPI) network was constructed by retrieving the interaction of the common DEGs from STRING database (Figure 4). The PPI network consists of 94 nodes (7 nodes from the common DEGs) and 97 edges. This PPI analysis revealed seven hub proteins, namely PDE9A, GNAO1, DUSP16, NTRK2, PGAM2, MAG, and TXLNA (Table 2).

### 3.3. Identification of Transcriptional Regulators of AD and IS

The gene expressions are controlled at transcriptional and post-transcriptional levels. To identify the transcriptional and post-transcriptional regulatory components of the common DEGs between IS and AD, we identified the significant TFs SPIB (targeting DEGs KRT78; DUSP16; PDE9A; KLHDC9), SMAD3 (targeting DEGs PDE9A), and SOX2 (targeting DEGs MAS1) (Table 2).

### 3.4. Protein–Drug Interactions of Common DEGs of AD and IS

**Figure 5:**
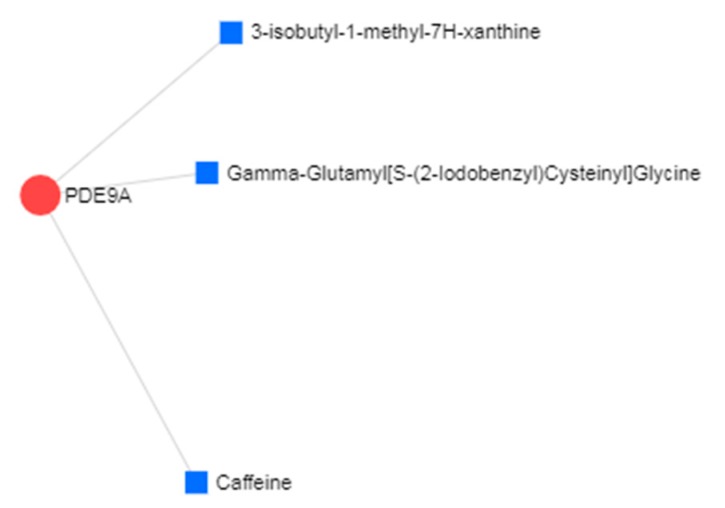
Protein–drug interactions network. The interactions between drugs and hub node (TUBB) were represented. The area of the node represents the degree of interaction in the network.

We studied the protein–drug interactions analysis and found that PDE9A protein has known interactions with three characterized compounds namely caffeine, γ-glutamyl [S-(2-iodobenzyl) cysteinyl]glycine, and 3-isobutyl-1-methyl-7H-xanthine (Figure 5).

## 4. Discussion

In the present work, we studied gene expression data of AD and IS patients in order to identify genes dysregulated in both diseases that may be candidate disease biomarkers and potential therapeutic targets. The candidate biomarker genes were explored in microarray and RNA-seq studies of AD and IS, an approach now commonly used to identify interactions between complex diseases [14,15,18,19,25,26]. Our analysis of these gene expressions patterns seen in AD and IS patients revealed numerous significant alterations, with expression profiles of 26 transcripts (22 unique genes) (termed “common DEGs”) that were found to be dysregulated in both transcriptomic datasets. The Gene Ontology and pathways analysis revealed a number of neurodegenerative disease associated pathways, i.e., alcoholism [27,28] and MAPK signaling pathway [29,30]. Nevertheless, this integrative analysis has the potential to identify novel key or hub genes that are common to these two diseases suggesting new lines of enquiry for studies, including identification of new targets for possible therapeutic intervention. Since both involve damage to the central nervous system, processes and dysregulated genes that they have in common are likely to be particularly important to that damage.

Protein–protein interaction analysis is increasingly used to discern important disease associated signaling molecules and pathways that may drive aspects of a disease. Thus, we analyzed PPI analysis of the corresponding proteins encoded by the AD- and IS-common DEGs to identify key or hub proteins with the potential to contribute to the progression of AD and IS (Table 2). Of those identified, phosphodiesterase-9 (PDE9) is already a potential target for treatment of the AD with phase II clinical trials underway for one PDE9 compound, BI409306, although this may not proceed to phase III but may be used for schizophrenia trials [31]. Mutations in *GNAO1* are already noted to be associated with neurologic pathophysiology [32]. DUSP16 is associated with neurological functions in axonal degeneration [33]. Chen et al. have reported that the genetic variants of NTRK2 is suggested to show a significant association between NTRK2 with AD [34]. Diseases associated with PGAM2 include glycogen storage disease X and phosphoglycerate mutase deficiency. Among their related pathways is central carbon metabolism in cancer and metabolism according to GeneCards database (www.genecards.org). Diseases associated with MAG include spastic paraplegia, autosomal recessive, and chronic polyneuropathy according to GeneCards database (www.genecards.org). Diseases associated with TXLNA (Taxilin Alpha) include B-Cell growth factor and inclusion conjunctivitis according to GeneCards database (www.genecards.org).

In addition to these protein interactions, we also identified several transcriptional regulators i.e., TFs that play a role in the functions of these common DEGs. The alteration in these molecules may provide critical information regarding the clarification of roles of biomolecules behind the diseases. Diseases associated with SPIB (Spi-B transcription factor) include primary biliary cholangitis and colorectal cancer according to GeneCards database (www.genecards.org). The relationship between tau hyperphosphorylation and TGF-ß1 signaling has also been recently studied in the temporal lobe in AD [35]. Interestingly, NFT protein can sequester phosphorylated Smad3 in AD brain, thus preventing its translocation into the nucleus and the induction of gene transcription [36,37]. Sox2 deficiency causes neurodegeneration and impaired neurogenesis in the adult mouse brain [37]. 

Finally, we studied the known protein–drug interactions to identify candidate drugs that may have the potential to influence AD and IS. Three compounds were identified from the interaction network. These require proper validation to determine their value, but this highlights that the present study provides insights into biomolecules and possible new avenues for therapeutic interventions, in addition to the potential for biomarker discovery for AD and IS.

## 5. Conclusions

In the present study, we analyzed gene expression transcriptomics profiles using systems biology analyses to reveal potential biomarkers that may clarify important pathobiological mechanisms underlying AD and IS. Significantly altered molecular pathways included alcoholism, MAPK signaling pathway, and glycine, serine, and threonine metabolism. We also detected seven significant hub proteins: PDE9A, GNAO1, DUSP16, NTRK2, PGAM2, MAG, and TXLNA. TFs that influence expression in the common DEGs was also identified, namely SPIB, SMAD3, and SOX2. These biomolecules may be considered systems biomarkers at the protein level and RNA levels. A number of compounds were identified from protein–drug interaction networks that might be investigated for their ability to block processes important to AD and IS. In sum, we have identified potential common biomarker signatures for AD and IS that may reveal new aspects of development and progression in these diseases.

## Figures and Tables

**Table 1:** The significant Gene Ontology terms (top five) enriched by the differentially expressed genes.

Category	Gene Ontology Term	*p*-Value
Biological process	Regulation of axonogenesis	0.002
	Learning or memory	0.006
	Brain development	0.007
	Behavior	0.009
	Aging	0.009
	Carbohydrate biosynthetic process	0.010
Cellular component	Neuron projection	0.014
	Cell projection part	0.014
	Axon	0.018
	Cell projection	0.019
Molecular function	Phosphoric ester hydrolase activity	0.004
	3′,5′-cyclic nucleotide phosphodiesterase activity	0.023
	3′,5′-cyclic nucleotide phosphodiesterase activity	0.023
	Phosphatase activity	0.026
	Hydrolase activity, acting on ester bonds	0.027

**Table 2:** A list of common biomarker candidates proposed for AD and IS in the present Study.

Biomarker Candidate	Name	Biological Roles/Significance of the Biomolecules
***Hub proteins***		
PDE9A	Phosphodiesterase-9	Already a potential target for treatment of the AD with phase II clinical trials underway for one PDE9 compound, BI409306
GNAO1	Guanine Nucleotide-Binding Protein, Alpha-Activating Activity Polypeptide O	Mutations in *GNAO1* are already noted to be associated with neurologic pathophysiology
DUSP16	Dual Specificity Phosphatase 16	Associated with neurological functions in axonal degeneration
NTRK2	Neurotrophic Receptor Tyrosine Kinase 2	The genetic variants of NTRK2 are suggested to show a significant association between NTRK2 with AD
PGAM2	Phosphoglycerate Mutase 2	Diseases associated with PGAM2 include glycogen storage disease X and phosphoglycerate mutase deficiency
MAG	Myelin Associated Glycoprotein	Diseases associated with MAG include spastic paraplegia, autosomal recessive, and chronic polyneuropathy
TXLNA	Taxilin Alpha	Diseases associated with TXLNA include B-cell growth factor and inclusion conjunctivitis
***Transcription Factor***		
SPIB	(Spi-B Transcription Factor)	Diseases associated with SPIB include primary biliary cholangitis and colorectal cancer
SMAD3	SMAD Family Member 3	NFT protein can sequester phosphorylated Smad3 in AD brain, thus preventing its translocation into the nucleus and the induction of gene transcription
SOX2	SRY-Box2	Sox2 deficiency causes neurodegeneration and impaired neurogenesis in the adult mouse brain
